# Progress in Diseases of the Blood

**Published:** 1902-05-03

**Authors:** 


### Progress in Diseases of the Blood.

**Iodophilia.**—A test for pneumonia has been found which is available before the physical signs appear and will doubtless be valuable, though it is also produced by suppuration and a few serious diseases of the blood which can be excluded by their own special symptoms. The test is applied as follows:—A blood-smear is dipped in a solution containing 3 per cent. of potassium iodide, 1 per cent. of iodine, and enough gum arabic to make it of the consistence of syrup. If the disease is present the protoplasm of the polynuclear cells is stained a reddish brown, either uniformly or so as to form a network, or finally as showing a number of pink or red granules. The reaction, according to T. Dunham,[1] varies with the intensity of the disease.

**Blood Counting.**—Lenhartz[2] recommends for rapid "fixing" a solution of formaldehyde 1 part, water 10 parts, methyl alcohol 89 parts. If the blood film is immersed in this for one minute perfect fixing may be obtained. Nucleated red cells are never met with in normal blood, except in the newly born. They are usually found in pernicious anæmia and leucæmia, and rarely in chlorosis or after great hæmorrhages; megaloblasts only in the embryo and in pernicious anæmia and leucæmia. Dwarf cells are found usually after hæmorrhages, and non-nucleated giant cells or megalocytes are found in pernicious anæmia and leucæmia and rarely in chlorosis. Among the white cells he notices that the polynuclear ones, whether neutrophile or eosinophile are actively motile on the warm stage, while marrow cells are motionless. In pernicious anæmia a few observers have rarely found actively moving infusoria sometimes armed with flagellæ. Kent and Calhoun[3] have made a study of the blood *platelets* which they regard as independent elements, but not hæmatoblasts in Hayem's sense. From elaborate experiments they find that they are the only elements which are disintegrated in the coagulation of normal blood. They are present to the number of 770,000 per cb. m. in health and bear a fixed ratio to the number of red cells. To count them it is desirable to prick the finger through a drop of fixing solution such as a 2½ per cent. formaldehyde in sodium chloride solution. *Granular degeneration of red cells* has been for some time a debatable subject. This symptom refers to the appearance of fine or coarse granules, either clumped or diffused, which have an affinity for basic stains, in red cells which may or may not be otherwise normal. White and Pepper[4] have reinvestigated them and find that they are constantly present in cases of lead poisoning, even at an early stage, and in persons working in lead in whom no other symptoms exist. They gradually disappear as convalescence is brought about. It is also easy to produce them in dogs to whom lead is administered, and in this case they can be detected a few days after the beginning of the experiment. The writers conclude that the granules represent a degeneration of the cell and have no relation to fragmentation of the nucleus or to polychromatophilia. Any ordinary stain may be used, such as hæmatoxylin and eosin. In this stain the hæmatoxylin should be in excess.

**Cryoscopy.**—The freezing point of blood as an aid to prognosis seems likely to be valuable. Normal blood, says A. Ogston,[5] freezes at 0·56° centigrade, but if elimination is defective, so that the blood contains an excess of impurities, this point is lowered to 0·58°, or even 0·71. Blood which freezes between 0·55° and 0·57° may be regarded as normal. The test is of importance to the surgeon who wishes to know whether the kidneys are acting sufficiently to justify an operation and its accompanying strain, or whether one kidney is strong enough to take on the whole work if its fellow is removed. Again, we can thus estimate the degree of danger in hepatic and other diseases. The calculation is made by Beckmann's freezing point apparatus, which consists of a jar of ice and salt, in which a large test tube is placed. A smaller tube within the large one contains the fluid to be tested, and a special thermometer, having a range of only six degrees and a zero point which can be varied. This zero represents the freezing point of distilled water, and must first be ascertained, after which the instrument must not be shaken or moved roughly for fear of displacing the adjustment. The blood to be tested is drawn from any large vein in the arm. The mercury falls first below the freezing point, then rises at the moment of congelation, and within a few minutes attains a steady maximum which is the true freezing point. The freezing-point of the urine has also been tested in this way, but the results are less satisfactory than those obtained from the blood, and no more definite than can be got from simply taking the specific gravity. The writer gives notes of 12 cases where the freezing-point of the blood was taken to make a prognosis. In four of these the point was found to be above normal, two of the four were neither anæmic nor hydræmic, but what is the cause of this unusual purity of the blood is not clear, though there is some reason to think it is pathological. The production of hæmoglobinuria has been hitherto a mystery, though it has been known for some time that various pathogenetic organisms such as the B. typhosus have a blood-destroying power which is, however, one of the feeblest of their effects. C. Todd[6] shows that the B. megatherium is powerfully hæmolytic without causing death or serious illness in certain animals. The lysin can be obtained separately by filtering strong cultures, and in large doses is fatal to guinea pigs, but in smaller ones they survive, though hæmoglobinuria is produced by it. If antiserum is injected at the same time or on a previous day no effect follows, showing that its action is not a simple chemical or physical one. The existence of such a power in a widely spread organism which usually produces no other symptoms is suggestive of the mode in which blood destruction takes place, in blackwater fever, paroxysmal hæmoglobinuria and pernicious anæmia.

**Pernicious Anæmia.**—Kormoczi[7] criticises the current rules of diagnosis as laid down by Ehrlich, especially as to the invariable presence in the blood of macrocytes and megalocytes. In five fatal cases which he examined there were no megaloblasts or nucleated red cells in the blood during life. In all of them he found macrocytes, megalocytes and megaloblasts in the marrow *post-mortem*. He believes that the blood during life cannot always be distinguished from that of severe secondary anæmia, but that the marrow is always of the megaloblastic type. Macrocytes are more characteristic and numerous in the blood than megaloblasts, but they are not diagnostic of the disease. W. Bain recently showed from some analyses the increase of the conjugated sulphates in the urine as well as of indican and urobilin, but he adds that the dark brown colour of the urine is not due to urobilin, for it remains after the urobilin has been extracted. Probably it is some derivative of hæmoglobin. A. McPhedran[8] thinks that both the common theories of origin are partly true, and that the disease is due both to blood destruction in the portal system and to defective blood formation from a common toxin, which also produces the nerve changes. From a study of 22 cases under his care he thinks that the early occurrence of marked weakness is also due to the toxin, and not to the anæmia. Indeed, the general condition bears little relation to the poorness of the blood. Some patients with very few red cells are able to do a surprising amount of work ; even the gastric intestinal symptoms may not occur till late. He finds that marked gastric changes or affections of the gums and teeth are not so frequent as we should expect if the disease originated here. The use of intestinal antiseptics has proved ineffective in his cases, and the same may be said of injections of various sera. Arsenic, strychnia, and purgation, indeed, lead to no better results in the long run, though the remissions which occur spontaneously in the disease may give a state of almost perfect health for a time irrespective of treatment. A. Goodall[9] takes the extreme view that the disease is due to an exhaustion of the blood-producing function from overwork. This may follow the long continuance of various morbid conditions, and when the degeneration is well established permanent recovery is hopeless. Thus he thinks the condition may follow and succeed to secondary anæmias.

[1] Med. Rec., Oct. 12. [2] Post Graduate. Oct. [3] Brit. Med. Jour., Nov. 23. [4] American Jour. Med. Science, Sept. [5] Lancet, Nov. 9. [6] Lancet, Dec. 14. [7] Med. Rec., Jan. 28. [8] Lancet, Jan. 18. [9] Scottish Med. and Surg. Jour., Jan.

